# Into the wild frontier: Mapping the terrain of adverse events in psychedelic-assisted therapies

**DOI:** 10.1177/02698811241292944

**Published:** 2024-10-29

**Authors:** Otto Simonsson, Simon B Goldberg, Peter S Hendricks

**Affiliations:** 1 Department of Neurobiology, Care Sciences, and Society, Karolinska Institute, Sweden; 2 Department of Counseling Psychology, University of Wisconsin—Madison, Madison, WI, USA; 3 Department of Psychiatry and Behavioral Neurobiology, University of Alabama at Birmingham, Birmingham, AL, USA

**Keywords:** Adverse events, psychedelics, commentary

The administration of psychedelics,^1^ in conjunction with psychotherapy (i.e., psychedelic-assisted therapy (PAT)), has shown promise as a potential treatment modality for various psychiatric disorders (Nutt and Carhart-Harris, 2021). For instance, in the United States, the Food and Drug Administration (FDA) has granted breakthrough therapy designation to psilocybin, as well as a deuterated psilocybin analog, combined with psychotherapy for the treatment of treatment-resistant depression and major depressive disorder (COMPASS Pathways, 2018; Cybin, 2024; Usona Institute, 2019), as well as lysergic acid diethylamide (LSD)-assisted therapy for the treatment of generalized anxiety disorder (MindMed, 2024). The same breakthrough therapy designation has also been given to 3,4-methyl enedioxy methamphetamine (MDMA)-assisted therapy for the treatment of post-traumatic stress disorder (MAPS, 2017), but the FDA recently reviewed the data submitted by the applicant, Lykos Therapeutics, and declined to approve MDMA-assisted therapy for the treatment of post-traumatic stress disorder in August 2024 (Lykos Therapeutics, 2024), partially due to safety concerns. The process of FDA approval of new treatments rests on a risk–benefit assessment (i.e., known and potential benefits vs known and potential risks), which requires knowledge of the full range of adverse events (AEs) that may be associated with the treatment. However, because psychedelics appear to be synergistic with psychotherapy (e.g., Levin et al., 2024) and are unique in that they reliably induce experiences characterized as spiritual, existential, religious, and theological (Palitsky et al., 2023), they could potentially be associated with distinct risks that distinguish them from traditional interventions. Even if these factors do not play into the FDA’s decision-making, it might nevertheless be important, especially for healthcare professionals, to develop a tailored assessment of AEs associated with PATs.

Palitsky and colleagues’ (2024) framework for the assessment of AEs occurring in PATs is both timely and important, especially considering the FDA’s recent decision to not approve MDMA-assisted therapy for the treatment of post-traumatic stress disorder. The authors of the framework formed a multidisciplinary working group, with diverse relevant experiences, that identified 54 potential AEs relevant to PATs and developed recommendations for their assessment across different phases. Palitsky et al. (2024) should be commended for their thoughtful consideration of the range of potential AEs that might be associated with PATs, drawing on the nascent psychedelic literature, as well as the more extensive literature on pharmacotherapy and psychotherapy. This framework will hopefully lead to standardized assessments of AEs in PATs that contrast with the inconsistency and lack of specificity of previous assessments (Breeksema et al., 2022).

The authors acknowledge the need for empirical validation and potential modification of their framework in future studies, but other limitations should be highlighted, such as the assessment burden. For instance, in addition to the AEs that are typically assessed in pharmacotherapy and psychotherapy trials, the framework includes AEs that are potentially unique to PATs. This may result in an extensive assessment of AEs that while potentially feasible for certain research contexts, may be too burdensome for researchers and participants alike in other research contexts, such as implementation studies or pragmatic trials. Ultimately, rather than assessing all possible AEs associated with PATs, it might be helpful to identify the most clinically relevant AEs and develop shorter assessments around these that can be reasonably used in practice.

Another issue worth considering is the possibility that assessments of AEs may themselves produce iatrogenic effects. For example, previous research suggests that if researchers intimate or suggest that certain AEs could occur, it increases the likelihood that participants will report those same AEs (Colloca and Barsky, 2020). It is possible that such nocebo effects could be further amplified by psychedelic-induced suggestibility (Carhart-Harris et al., 2015), which highlights the need for strategies to minimize these effects. This issue may be avoided to some degree by balancing the number of potential benefits and risks of PATs presented to participants, using clinician- and informant-based reports of AEs, or by measuring and controlling for patients’ expectations of AEs. Other research indicates that nocebo effects could potentially also be countered by explaining the nature of nocebo effects to participants (Pan et al., 2019). This could be part of the informed consent process in PATs.

Because it is not yet empirically known whether and to what degree PATs increase the risk for AEs delineated by Palitsky et al. (2024), it will be important to conduct randomized trials that compare the prevalence of AEs in PATs to both placebo and other treatment modalities, including those that do not involve the administration of a psychedelic. Such studies should be conducted with different types of psychedelics and in various patient populations. This would help to elucidate which, if any, AEs are unique to PATs and whether there are differences across types of psychedelics and patient populations.

In conclusion, by addressing the need for a standardized assessment framework for AEs in PATs, Palitsky and colleagues’ (2024) framework represents a significant step forward in the field of psychedelic research, which may contribute to more robust scientific evidence and better patient outcomes should these treatments be approved. While the proposed framework is comprehensive and well-structured, further empirical validation and potential modification of the framework are needed to ensure its effectiveness and practicality. Future studies should investigate the relative prevalence of AEs across treatment modalities and consider ways to balance thorough assessment of AEs without increasing the risk for iatrogenic effects from the assessments themselves.

## Notes

1. Because this is a commentary of the manuscript written by Palitsky et al. (2024), we adopted their definition of psychedelics: classic psychedelics (e.g., psilocybin, LSD, ayahuasca), MDMA, ibogaine, and other drugs that are described as inducing psychedelic-like experiences (e.g., ketamine).
